# Copy Number Variation of the CADM2 Gene and Its Association with Growth Traits in Yak

**DOI:** 10.3390/ani9121008

**Published:** 2019-11-21

**Authors:** Fei Ge, Congjun Jia, Min Chu, Chunnian Liang, Ping Yan

**Affiliations:** Key Laboratory of Yak Breeding Engineering Gansu Province, Lanzhou Institute of Husbandry and Pharmaceutical Science, Chinese Academy of Agricultural Sciences, Lanzhou 730050, China; 82101185176@caas.cn (F.G.); dkjcj@nwafu.edu.cn (C.J.); chumin@caas.cn (M.C.)

**Keywords:** copy number variation (CNV), CADM2 gene, growth traits, association analysis, yak

## Abstract

**Simple Summary:**

Cell adhesion molecule 2 (CADM2), also known as synaptic cell adhesion molecule 2 (SYNCAM2), is the mediator of synaptic signals enriched in the brain. Overlaps between copy number variation (CNV) regions in CADM2 and quantitative trait loci (QTL) related to body weight have been clarified in a previous study. In this study, two loci were amplified in the CADM2 gene (CNV1: 235,915 bp, exon 1 and partial intron 1; CNV2: 60,430 bp, intron 9) to explore the relationship between CNV types in the CADM2 gene and growth traits in 350 Ashidan yaks. Association analysis illustrated that no significant effect was found on growth traits in CNV1. However, the CNV2 mutation had a significant effect on body weight at the sixth month (*p* < 0.05). Individuals with the gain-type copy number variation CNV2 were significantly superior to those with loss- or normal-type in terms of body weight (*p* < 0.05). In summary, this study confirmed that CADM2-CNVs affect growth traits in yaks, and may be candidate genes for successful yak breeding and genetics projects.

**Abstract:**

Copy number variation (CNV) is currently accepted as a common source of genetic variation. It is reported that CNVs may influence the resistance to disease and complex economic traits, such as residual feed intake, muscle formation, and fat deposition in livestock. Cell adhesion molecule 2 (CADM2) is expressed widely in the brain and adipose tissue and can regulate body weight through the central nervous system. Growth traits are important economic traits for animal selection. In this study, we aimed to explore the effect of CADM2 gene copy number variants on yak growth traits. Here, two CNVs in the CADM2 gene were investigated using the quantitative polymerase chain reaction (qPCR), and the association of the CNVs with growth traits in yak was analyzed using statistical methods by SPSS software. Differences were considered significant if the *p* value was < 0.05. Statistical analysis indicated significant association of CADM2-CNV2 with the body weight of the Chinese Ashidan yak. A significant effect of CNV2 (*p* < 0.05) was found on body weight at 6 months. In CNV2, the gain-type copy number variation exhibited greater performance than the other variants, with greater body weight observed at 6 months (*p* < 0.05). To the best of our knowledge, this is the first attempt to investigate the function of CADM2-CNVs and their association with growth traits in animals. This may be a useful candidate marker in marker-assisted selection of yaks.

## 1. Introduction

With large duplications and deletions in gene copy numbers detected in humans and other primates, it is not unreasonable to expect that copy number variation could be a significant source of genetic variation in other animals [1]. In previous studies, many molecular markers have been assessed for improving economic traits in animal breeding, including restriction fragment length polymorphisms (RFLP), single nucleotide polymorphisms (SNP), indels (Insertion/Deletion) and structural variations, such as inversions, duplications and deletions. To date, SNPs are used extensively for complex economic disease traits in livestock [2,3,4,5]. More than 99% of indels are less than 50 bp in length [6,7], and some long insertions and deletions are returned into the concept of structural variation [8]. As a genetic marker, indels have great potential whether in growth and reproduction traits or the treatment of disease [9,10,11]. However, CNVs include duplication and/or deletions of DNA fragments ≥50 kb, and can cause a greater number of differences than SNPs and indels [12], which can be discovered by clone-based array comparative genome hybridization (array-CGH) [13]. Copy number variants can be engendered through four mechanisms: non-allelic homologous recombination (NAHR), non-homologous end joining (NHEJ), fork stalling and template switching (FoSTeS), and L1-mediated retro-transposition [14,15]. This submicroscopic structural variation comprises insertions, inversions and translocations in the genome [13]. Compared with SNPs, CNVs have a higher rate of mutation, with a number of diseases and congenital deficiencies caused by the action or the process of mutation in CNVs. Increasing evidence indicates that CNVs are associated with a number of complex human diseases, for example, the susceptibility to HIV-1 [16], autism spectrum disorders [17] and Tourettes Syndrome [18]. Conversely, many studies in livestock have revealed that CNVs are correlated with some important traits, such as parasite resistance in Angus cattle [19], growth traits in Chinese cattle [20,21], meat production and quality in pig [22] and feed conversion ratios in beef cattle [23]. These results demonstrate that CNVs may have a great influence on variation in phenotype, probably because of the difference in the copy number of specific sequences.

The yak (*Bos grunniens*) is critical to populations living on the Qinghai-Tibet Plateau and adjacent regions, as they provide materials indispensable for survival such as meat, milk, transportation and fur [24]. The yak’s ability to survive in harsh conditions makes it an ideal model organism to explore the mechanism of adaptation to high elevations in both animals and human beings. The Ashidan yak, a new yak breed bred by Chinese scientists, has typical characteristics such as lack of horns and a mild temperament. It is easier to maintain and feed in the stalls, and thus suited for large-scale intensive breeding in the cold and arid alpine areas of China. In recent years, several functional CNV regions have been discovered and validated in the yak, utilizing an NGS-based method [25,26,27]. In addition to this, in a previous association study, CNVRs throughout the yak genome have been constructed using SNP arrays which indicate that the cell adhesion molecule 2 (CADM2, also known as SynCAM2, Igsf4d and Nectin-like molecule 3) gene overlaps with a portion of CNVR29 and CNVR30 [28]. Variants of the CADM2 gene have been previously identified to play a pivotal role in the human body mass index (BMI) values by means of the central nervous system [29,30,31]. Furthermore, analysis in mice has found that CADM2 is strongly linked to body weight and energy homeostasis via brain activity [32].

In addition, a previous study has demonstrated that the CADM2 gene was located in quantitative trait loci (QTLs) related to body weight both at weaning and in yearlings in cattle [33]. Together, this evidence indicates that CADM2-CNVs in yaks may play a vital role in growth traits that are useful in artificial selection. Nevertheless, research of CADM2-CNV has lagged far behind, in humans and other animals. We hypothesize that copy number variants in the CADM2 gene may affect yak phenotypic traits. The present study investigated the interrelations of CNVs including CNV1 (235,915 bp, exon 1 and partial intron 1) and CNV2 (60,430 bp, intron 9) with the CADM2 gene with growth traits in the yak, by performing quantitative real-time PCR (qPCR).

## 2. Materials and Methods

### 2.1. Ethics Statement

All experiments in the current research were approved by the Key Laboratory of Yak Breeding Engineering of Gansu Province, Lanzhou Institute of Husbandry and Pharmaceutical Sciences (No. LIHPS-CAAS-2017-115). Blood sample collection and body measurements were performed in strict accordance with the guide for the Care and Use of Laboratory Animals.

### 2.2. Sample Collection and Growth Traits

Three hundred and fifty unrelated female Ashidan yaks that had been raised with the same levels of nutrition were randomly selected from the Datong yak farm (Qinghai Province, China). None of the animals involved in this study were unhealthy. Phenotypic data (i.e., body weight, withers height, body length, chest girth) were measured in the selected Ashidan yaks at three stages of growth (i.e., at 6 months, 1 year and 18 months of age) for subsequent analysis. Phenotypic measurements were strictly in line with standard methods proposed by Gilbert et al. [34].

### 2.3. Genomic DNA Extraction and Copy Number Identification

Genomic DNA was extracted using an EasyPure Blood Genomic DNA kit (TransGen Biotech Co., Ltd., Beijing, China) and its concentration and quality measured using a Nanodrop 2000 spectrophotometer (ThermoFisher Scientific Inc., Waltham, MA, USA). Quantified DNA samples were stored at −20 °C.

**Figure 1:**
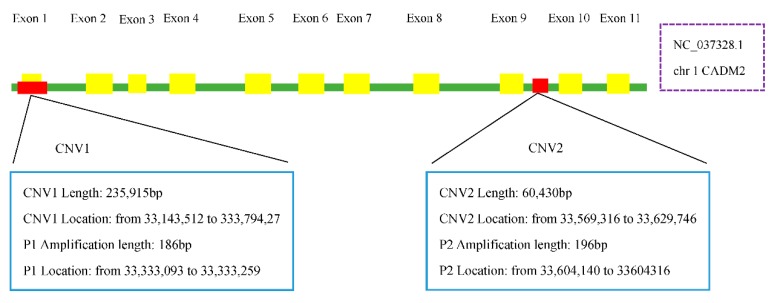
The two copy number variation regions of the CADM2 gene. The yellow boxes represent the coding regions and red boxes represent the CNVRs.

The copy number variation regions CADM2-CNV1 and CADM2-CNV2 are located at 33,143,512–33,379,427 bp and 33,569,316–33,629,746 bp from the reference genome sequence NC_037328.1, respectively (Figure 1).

### 2.4. Primer Design

The National Center for Biotechnology Information (NCBI) primer-BLAST web tool was utilized to design the primers for the analysis of CADM2-CNVs (Table 1). A polymerase chain reaction (PCR) was utilized to verify the quality and optimum temperature of primers. Products were analyzed on 1% agarose gel electrophoresis.

### 2.5. Quantitative PCR Analysis

A LightCyler^®^ 96 quantitative polymerase chain reaction (qPCR) system (Roche, Basel, Switzerland) with SYBR Green dye was used in this study because of its convenience and accuracy. The bovine basic transcription factor 3 (BTF3) gene was chosen as the diploid internal reference gene [35]. The total volume of the reaction mixture was 20 μL, comprising 40 ng genomic DNA with 10 μL of SYBR Premix Ex Taq II (TaKaRa, Dalian, China), 2 μL of amplification primers and 7 μL of ddH_2_O. The q-PCR procedure involved a 30 s preincubation at 95 °C then a two-step amplification with 45 cycles of 95 °C for 5 s followed by 62 °C for 30 s. After completion of the cycling, the reaction was terminated by incubation at 95 °C for 5 s then 65 °C for 60 s then 95 °C continuously. All experiments were conducted in triplicates, and the results represented as mean values ± standard deviation (SD).

### 2.6. Statistical Analysis

Copy number values of the yak CADM2 gene were determined using the formula: $2\times 2^{-\Delta \Delta ct}$ [36]. Cycle threshold (Ct) values for the target gene were normalized using the formula: $\Delta Ct=Ct_{target}-Ct_{reference}$, with ΔCt of the test samples normalized using the formula: $\Delta \Delta Ct=\Delta Ct_{test}-\Delta Ct_{control}$. As the precondition of the analysis of variance, the homogeneity of variance test and the normality test of each trait in different copy number variation (CNV) regions were done by Bartlett test and Shapiro-Wilk test before association analysis. One-way analysis of variance (ANOVA) and a nonparametric test were used to analyze the association of CADM2 gene copy number variant types with growth traits in the Ashidan yak. The following model was used to analyze the CNV effects on growth traits: $Y_{ij}=\mu +CNV_{ij}+e_{ij}$, where *i* represents the *i*th region, *j* the *j*th CNV type, $Y_{ij}$ the observed growth trait, μ the overall mean of each trait, with $CNV_{ij}$ representing the effect of *i*th CNV region and *j*th CNV type of the CADM2 gene. $e_{ij}$ is a random error. Assessment of differences between means was performed by one-way ANOVA with a post hoc least significant digit (LSD) multiple comparison test. Differences in the distribution of CNV types for different CNV regions were analyzed using a chi-square test in terms of the frequency of CNV types [37]. Based on the results of association analysis, a Kendall Tau correlation test was carried out. Other than these, the combination and interaction effect of CNV1 and CNV2 also detected by two-way analysis of variance (ANOVA).

All the statistical analyses were carried out using IBM SPSS Statistics software (Version 23, IBM, New York, NY, USA). Significance level was set up at *p* < 0.05 in this experiment.

## 3. Results

### 3.1. Quantitative PCR and the Distribution of CADM2-CNVs

**Figure 2:**
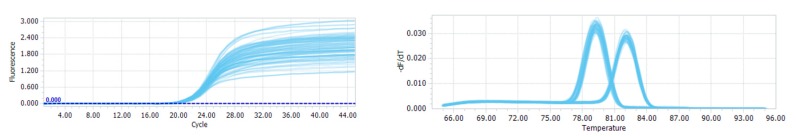
Amplification curves and melt peaks of the CADM2 and BTF3 genes.

**Figure 3:**
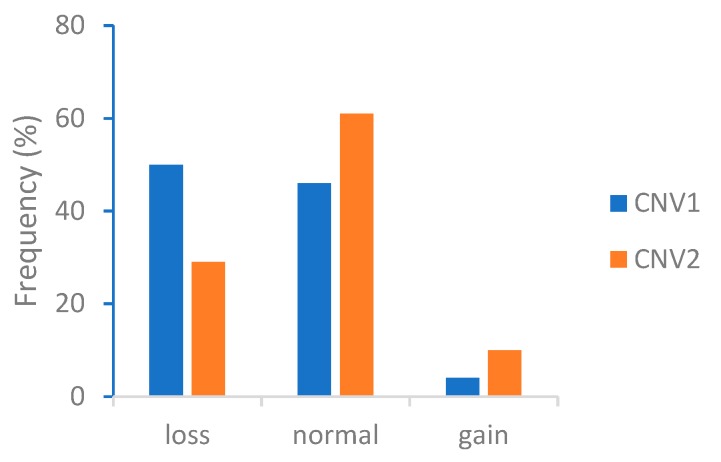
Distribution of different copy number variation (CNV) types in CNV1 and CNV2 in the Ashidan Yak.

Specific primers for the two CNVs are displayed in Table 1. Cycle threshold (Ct) values were used subsequently for quantitative analysis. The amplification curves and melt peaks (Figure 2) were used to determine primer specificity. In addition, the distribution frequency of different CNV types in CNV1 and CNV2 were measured (Figure 3 and Table 2).

The majority of individual animals had the loss-type variant in CNV1, the proportion exceeding 50%. However, in CNV2, the largest number of individuals had the normal variant, followed by the loss-type. The gain-type variant was similarly distributed within CNV1 and CNV2. The chi-square test indicated a significant difference in the distribution of CNV types between CNV1 and CNV2 (*p* < 0.01), suggesting that the CNV type is specific for different loci (Table 3).

### 3.2. Association Analysis

Association analysis indicated a significant correlation between copy number type and phenotypic measurements in the Ashidan yak population. Bartlett test showed the homogeneity of variance of each trait within each locus (Supplementary Table S1). And the Shapiro-Wilk test showed the normality of each trait in different CNV regions (Supplementary Table S2). One-way ANOVA indicated that the CNV2 mutation had a significant effect on body weight (*p* < 0.05) in 6th month yaks (Table 4 and Table 5). Multiple comparisons were conducted based on the ANOVA described above. Interestingly, the gain-type copy number variant of CADM2-CNV2 exhibited clear phenotypic superiority in comparison with the loss and normal types, with greater body weight values in 6th month Ashidan yaks (*p* < 0.05). Furthermore, our correlation test verified the significant correlation between CNV2 and body weight in 6th month (*p* < 0.01) (Table 6).

No significant difference was observed in the traits at 12 and 18 months. Association analysis of the CADM2 gene CNVs with growth traits is presented in Table 4 and Table 5.

Moreover, a two-way analysis of variance (ANOVA) was also carried out to test the joint effect of CADM2-CNV1 and CADM2-CNV2. The results revealed a significant association in 18th month withers height both in CNV1 (*p* < 0.01) and CNV2(*p* < 0.05) (Table 7). But no significant association was found in their combination.

## 4. Discussion

Researchers have previously given considerable attention to the analysis of molecular markers associated with economic traits in a number of species. For example, genetic variation in the ovine PROP1 gene was identified in 670 New Zealand Romney sheep, which were related to traits such as tailing weight and growth rate [38]. One 43-bp indel polymorphism in HS6ST3 was associated with shank length, chest depth and other growth traits in 860 chickens [39]. SNPs within the coding region of the KDM4D gene were explored and linked with testis morphology traits of male pigs [40]. However, copy number variance gives rise to more substantial mutations and more extensive genetic effects than other molecular markers [41]. It has been established that the total number of nucleotides affected by copy number variants is larger than the total number of SNPs [42]. Recently, CNV maps in cattle [35], sheep [43] and pigs [44] have been constructed, indicating that structural polymorphisms within them constitute an important proportion of their genomes. Furthermore, CNVs have been associated with various growth traits, such as body length in cattle [45] and pigs [46] and pigmentation traits in goats [47] and horses [48], in addition to reduced immunological resistance to diseases [49]. Consistent with these results, it is widely believed that this form of genetic variation in livestock is of great importance to phenotypic traits.

Quantitative polymerase chain reaction (q-PCR) is widely used, especially for the measurement of copy number variation because of its accuracy and simplicity. To investigate the probable effects of CADM2-CNVs in the yak growth process, we conducted an association study between 12 phenotypic traits and different CNV types using a general linear model for the first time. Significant association was observed between 6th body weight and CNV2. The statistics demonstrate that weaning weights are significantly correlated with CNV2 type. Interestingly, this is in agreement with the conclusion of previous studies [28]. This phenomenon may be due to the function of the CADM2 gene, which plays an important role in maintaining the synaptic circuitry of the central nervous system [50], and can mediate leptin sensitivity, thermogenesis and energy metabolism [51]. Notably, the gain-types in CNV2 caught our attention due to the outstanding body weight in 6th month animals. Here, no significant association between CNV1 and CNV2 and the body weight in 12 month-olds was observed, while in cattle a QTL harboring CADM2 gene was thought to be associated with the yearling weight. This was most likely cause by the following points. First of all, a quantitative trait like body weight is regulated by multiple genes with small effect. Though a 350 yak sample size is an appropriate sample to examine the variance, the marker’s effect is too insufficient to be detected. A larger population is needed to be studied in the future. Secondly, it is probably due to the yak’s special physiological regulation mechanism due to its particularity harsh living conditions. Unlike the cattle industry, yak has to struggle to survive in a cold season lasting for six months after weaning, which is extremely cold along with severe forage shortage. 

A 6 months hay period they will endure after weaning, which is different from cattle. This phenomenon may lead to a unique physiological regulation of growth in yaks. Here, we did not find any associations between CNV types and phenotype at 12 and 18 months.

To explain why this CNV has such an uncommon influence on phenotype, firstly the CADM2 gene had been reported to overlap a QTL region that is correlated with body weight (weaning and yearling) [28]. Secondly, body weight and energy metabolism are maintained by the central nervous system and previous studies have identified that CADM2 is expressed more abundantly in different regions of the brain than in any other tissue [52]. Furthermore, plenty of studies have reported that CADM2 can significantly improve body weight and decrease leptin sensitivity via the brain and adipose tissue [32,53]. Moreover, genome-wide association studies in humans have demonstrated clear evidence that the CADM2 gene is strongly associated with the body mass index through the encoding of membrane proteins that mediate synaptic assembly [31,52,54]. Because of the importance of both the CADM2 gene and copy number variance in animals, we propose that CADM2-CNVs can be used as indispensable molecular markers of genetic improvement of the yak. To the best of our knowledge, copy number variance of CADM2 has not been previously investigated in livestocks such as cattle, sheep, pigs, etc. Therefore, we speculate that the CADM2 gene might play a crucial role in growth traits in the yak.

Growth traits are an economically important factor in animal husbandry that can improve production performance by selective breeding [55]. Consistent with this research, numerous studies have illustrated the association between gene copy number type and growth traits. Shi et al. demonstrated that the CNV of the LEPR gene exhibited significant correlation with growth traits in Chinese cattle [56]. Jiang et al. clarified that CNV of the sheep SHE gene was greatly linked with economic traits, such as body length and chest width [57]. Lin et al. suggested that the CNV of SOX6 is responsible for muscle development in chickens [58]. We propose that these molecular markers in various livestock offer the theoretical promise of animal selection.

The yak is a specialized breed famous for its adaptation to life on the plateau and hypoxia. Distinctive regulatory mechanisms require exploration, particularly their growth mechanism. In the present study, the two CNVs of the CADM2 gene in the Ashidan yak have been characterized and validated. The results demonstrate the effect of the CADM2 CNVs on phenotypic traits in different CNV types. From this evidence, we have established that CADM2 CNVs can act as molecular markers in Ashidan yaks for early selection.

## 5. Conclusions

In conclusion, this study described the distribution of CADM2 gene copy number variant types in 350 Ashidan yaks. CADM2-CNV2 is significantly associated with body weight in 6 months in the Ashidan yak. Further studies, such as the mechanism of physiological regulation with the CADM2 gene in yaks need to be carried out. Also a larger population is required to validate the association of this CADM2 gene with growth traits in the yak.

## Figures and Tables

**Table 1:** Details of primers.

Gene	Primer Pairs Sequence (5′-3′)	Amplicon Length (bp)	Tm (°C)
CADM2-P1	F: GACTTCCCAGGATTGCCTGT R: CCCTGGGAGCACAGTTGTTT	186	62 °C
CADM2-P2	F: GGCTGTCACGTTCTTCTCTCA R: AGGGTTCATCCTGGAGGCTT	196	62 °C
BTF3	F: AACCAGGAGAAACTCGCCAA R: TTCGGTGAAATGCCCTCTCG	166	62 °C

F: forward primer; R: reverse primer.

**Table 2:** Distribution and frequency of CNV types in CNV1 and CNV2.

Type	Loss	Normal	Gain
CNV1	176 (0.50)	161 (0.46)	13 (0.04)
CNV2	101 (0.29)	215 (0.61)	34 (0.1)

Figures represent numbers of animals (and proportion of the total population studied).

**Table 3:** Chi-square test of CNV type distribution in CNV1 and CNV2.

Type	CNV1	CNV2
CNV1		37.445 (*p* = 0.000 **)
CNV2		

Chi-square values ($\chi^{2}$) for differences between CNVs. ** Represent statistically significant differences at a level of *p* < 0.01.

**Table 4:** Statistical analysis of CADM2-CNV1 growth traits in the Ashidan yak.

Age	Growth Trait	CNV Type (Mean ± SE)			*p*-Value
		Loss (0.50)	Normal (0.46)	Gain (0.04)	
6 months	Body weight (kg)	83.14 ± 0.773 ^a^	85.60 ± 0.833 ^b^	81.15 ± 2.207 ^a,b^	0.052
	Withers height (cm)	94.50 ± 0.376	94.27 ± 0.435	95.31 ± 1.491	0.765
	Body length (cm)	91.46 ± 0.557	92.53 ± 0.594	90.54 ± 1.228	0.325
	Chest girth(cm)	124.21 ± 0.567	123.66 ± 0.662	124.38 ± 1.607	0.802
12 months	Body weight (kg)	82.17 ± 0.784	83.14 ± 0.881	80.64 ± 3.058	0.584
	Withers height (cm)	90.62 ± 0.324	90.28 ± 0.335	90.31 ± 0.894	0.755
	Body length (cm)	95.98 ± 0.380	95.74 ± 0.387	96.92 ± 1.719	0.682
	Chest girth(cm)	117.35 ± 0.379	116.93 ± 0.428	116.92 ± 0.843	0.747
18 months	Body weight (kg)	121.82 ± 1.119	122.59 ± 1.258	126.25 ± 3.371	0.516
	Withers height (cm)	101.71 ± 0.452	101.73 ± 0.476	105.54 ± 3.098	0.180
	Body length (cm)	101.22 ± 0.464	102.07 ± 0.460	101.69 ± 1.784	0.439
	Chest girth(cm)	137.97 ± 0.840	138.57 ± 0.795	141.54 ± 3.580	0.464

^a,b^ Represent statistically significant differences at a level of *p* < 0.05.

**Table 5:** Statistical analysis of CADM2-CNV2 growth traits in the Ashidan yak.

Age	Growth Trait	CNV Type (Mean ± SE)			*p*-Value
		Loss (0.29)	Normal (0.61)	Gain (0.1)	
6 months	Body weight (kg)	82.22 ± 1.004 ^a^	84.75 ± 0.723 ^b^	86.61 ± 1.559 ^c^	0.048 *
	Withers height (cm)	94.27 ± 0.544	94.28 ± 0.357	95.82 ± 0.785	0.263
	Body length (cm)	90.93 ± 0.743	92.30 ± 0.499	92.74 ± 1.270	0.278
	Chest girth(cm)	123.56 ± 0.877 ^a^	123.66 ± 0.518 ^a^	127.08 ± 1.013 ^b^	0.051
12 months	Body weight (kg)	82.53 ± 1.107	82.43 ± 0.726	84.53 ± 2.013	0.579
	Withers height (cm)	90.09 ± 0.456	90.54 ± 0.278	90.94 ± 0.729	0.534
	Body length (cm)	95.64 ± 0.491	96.09 ± 0.333	95.47 ± 1.038	0.656
	Chest girth(cm)	116.74 ± 0.525	117.24 ± 0.355	117.65 ± 0.778	0.606
18 months	Body weight (kg)	122.71 ± 1.510	121.71 ± 1.076	125.19 ± 1.996	0.428
	Withers height (cm)	101.49 ± 0.640	102.31 ± 0.414	100.34 ± 1.299	0.180
	Body length (cm)	101.11 ± 0.667	101.79 ± 0.396	102.13 ± 0.913	0.567
	Chest girth(cm)	137.99 ± 1.165	138.45 ± 0.698	139.19 ± 1.923	0.844

Association of CNVR30 of the CADM2 gene with yak growth traits. ^a,b,c^ Represent statistically significant differences at a level of *p* < 0.05; * *p* < 0.05.

**Table 6:** Correlation test of body weight in 6 months and CNV2.

Kendall Tau correlation coefficient	0.261
Significance	0.007 **

** Represent statistically significant differences at a level of *p* < 0.01.

**Table 7:** Two-way analysis of variance (ANOVA) of the effects of CADM2-CNVs in the Ashidan yak.

Age	Growth Trait	CNVs (*P* Value)		
		CNV1	CNV2	CNV1 × CNV2
6 months	Body weight (kg)	0.129	0.485	0.884
	Withers height (cm)	0.439	0.295	0.418
	Body length (cm)	0.470	0.960	0.370
	Chest girth(cm)	0.905	0.652	0.387
12 months	Body weight (kg)	0.447	0.958	0.859
	Withers height (cm)	0.870	0.944	0.735
	Body length (cm)	0.631	0.891	0.996
	Chest girth(cm)	0.997	0.995	0.845
18 months	Body weight (kg)	0.622	0.941	0.354
	Withers height (cm)	0.003 **	0.039 *	0.190
	Body length (cm)	0.508	0.821	0.750
	Chest girth(cm)	0.122	0.253	0.168

* Represent statistically significant differences at a level of *p* < 0.05; ** Represent statistically significant differences at a level of *p* < 0.01.

## Supplementary Materials

The following are available online at https://www.mdpi.com/2076-2615/9/12/1008/s1, Table S1: The homogeneity of variance test of each trait, Table S2: The test of normality of each trait in different CNV regions.
